# A focus on the discovery of potent and selective cyclic peptide scaffolds for drug development

**DOI:** 10.1039/d2sc90214a

**Published:** 2022-11-04

**Authors:** George J. Saunders, Andrei K. Yudin

**Affiliations:** a Davenport Research Laboratories, University of Toronto 80 St. George St Toronto M5S 3H6 Ontario Canada andrei.yudin@utoronto.ca

## Abstract

In the past, cyclic peptide drugs were commonly discovered by isolation of natural products. However, recent efforts predominantly use high-throughput synthetic or genetically encoded libraries to find potent and selective hits against a range of challenging therapeutic targets. Kawamura *et al.* (*Chem. Sci.*, 2022, **13**, 3256–3262, https://doi.org/10.1039/D1SC06844J) developed a new workflow that can be applied to mRNA display, using high-throughput clustering, SAR investigations and *in silico* structural studies. This led to the discovery of nanomolar, serum-stable cyclic peptides against the human glucose-dependent insulinotropic peptide receptor (hGIP-R).

## Abstract 2

Library-based screening methods such as mRNA display are paving the way for the discovery of cyclic peptides towards previously undruggable space.

## Graphical abstract

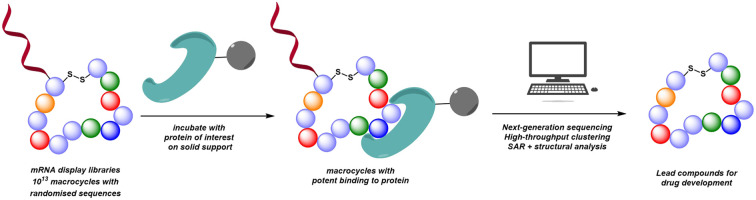

Cyclic peptides are a unique class of compound that fit into a niche area of chemical space between small molecules and larger biomolecules such as antibodies. Their large polar surface areas allows them to act in a selective and potent manner against challenging intracellular targets, such as protein–protein interactions (PPIs), which are often inaccessible to small molecules.^1^ In addition, a constrained backbone benefits from a lower entropic cost upon binding compared to acyclic congeners.

Traditional medicinal chemistry efforts towards the development of cyclic peptides have focused on the isolation of natural products.^2^ For example, the immunosuppressant drug cyclosporin A was isolated from the fungus *Tolypocladium inflatum* in the early 1970s and entered the clinic in 1983. It is a macrocyclic undecapeptide containing 33 atoms in its backbone, weighing in at just over 1200 Da. Remarkably, a molecule of this size displays excellent bioavailability, despite existing far beyond the rule of five (bRo5).^3^ However, controlling the passive permeation and oral bioavailability of cyclic peptide scaffolds is a difficult and enduring challenge in the development process for this class of molecule.^4^

To emulate cyclosporin A, chemists often look to incorporate backbone modifications: *N*-methylated and d-amino acids, as well as other synthetic modifications such as heterocycles or lipophilic groups.^5^ Moreover, a rich array of diversity can be added to amino acid side chains using canonical or unnatural amino acid building blocks. New advances in synthetic chemistry have enabled structure–activity relationships to be carried out in macrocycles akin to small molecules, enabling fine-tuning of the physicochemical properties, biological activity and target selectivity of potential macrocyclic drug candidates.^6^

Nowadays, the modern drug discovery process for macrocyclic peptides is dominated by library-based screening methods.^7,8^ The key advantage of this strategy is that millions, billions or even trillions of diverse macrocyclic scaffolds can be generated and screened against specific targets. A chemical synthesis method can be employed, with strategies such as DNA encoded libraries (DEL) and one-bead-one-compound approaches that rely on split-and-pool techniques using small building blocks to iteratively add diversity to the library.^9^ A fundamental aspect of DEL is that each building block is accompanied by a unique DNA tag that can be sequenced in the end to discover the identity of the hit compound. Although these methods can be carried out with small amounts of building blocks, if a complex unit forms part of the molecule of interest, it can be challenging to re-synthesise the target in a timely manner.

Macrocycles can also be made using the transcription and translation machinery present in cells to produce genetically encoded cyclic peptide libraries.^10^ Importantly, the cyclic peptides are linked to the mRNA or DNA that encodes them, which allows for the identity of the macrocycle to be determined. There are three main methods: phage display, split-intein circular ligation of peptides and proteins (SICLOPPS), and mRNA display.^11^ While there are advantages and disadvantages to each method and they have all found success in the discovery of potent macrocyclic compounds, mRNA display can be used to install unnatural, non-proteinogenic amino acids and modified building blocks. In this way, chemists can produce molecules inspired by natural products which have a chance to possess suitable pharmacokinetic properties.^12^

Predictably, for a method generating so many compounds, there is a staggering amount of data that comes from mRNA display libraries. The arrival of next-generation sequencing (NGS) has enabled chemists to collect vast data sets compared to classical Sanger sequencing. As modern drug discovery relies more and more on mRNA display techniques, it is vital that new high-throughput data processing methods and workflows are found that take advantage and extract the most out of this information. Typically, only the most abundant sequences in the library are determined and developed, but this does not discriminate between useable hits and false positives that have non-specific binding.^10^ However, recent work presented by Kawamura *et al.*^13^ introduces an improved workflow that can be used with mRNA display to streamline the development process and find functional hits in a more efficient manner.

In a collaborative effort, the Kawamura group (University of Oxford) and a team of chemists at Novo Nordisk, sought to discover potent, selective and GIP-ligand competitive macrocyclic peptides to inhibit the type 2 GPCR glucose-dependent insulinotropic peptide receptor (GIP-R), which has been studied as a potential target for type 2 diabetes and obesity.

An mRNA display library was designed, which contained sequences containing six to fourteen random amino acid residues, including two conserved cysteine residues at each end that form disulphide-bridged macrocycles. In this way, trillions of macrocycles with randomised sequences were generated and screened against the biotinylated extracellular domain of the human GIP-R (hGIP-R ECD) immobilised on a solid support. After five rounds of library enrichment, NGS of the complementary DNA from the mRNA library was carried out, and the sequences underwent high-throughput clustering organised by similarity to give thirteen individual clusters composed of the top 3160 hits. The most abundant and diverse peptides were selected from each cluster and prepared using parallel solid-phase peptide synthesis (SPPS).

With a set of macrocycles in hand, the binding of the individual peptides was determined using biolayer interferometry (BLI). Staggeringly, greater than 50% of the selected sequences had nanomolar affinity for hGIP-R ECD. Although many of the most abundant peptides were potent binders, some of the sequences from clusters that featured low abundance were also strong binders. This demonstrates the utility of the clustering technique, which helped to give a fuller assessment of the sequences within the library, not just based on their overall abundance.

At this stage, although it was known that the macrocycles within the library could bind to hGIP-R ECD, information about the specific binding site or whether the ligands were competitive with GIP had not been determined. To establish this, competitive displacement assays with ^125^I-GIP were performed. None of the most abundant and high affinity sequences, covering over 50% of the entire dataset, showed competitive binding with ^125^I-GIP. In fact, only peptides from two specific clusters displaced ^125^I-GIP from the receptor, both containing a conserved tetrapeptide motif within their sequence. This further highlights the strength of the clustering technique, which enables the discovery of highly functional molecules rather than just selecting the most abundant sequences within the library (which may not show competitive binding *versus* a biological ligand).

Now that the clusters containing competitive binders had been found, they could be further explored to find other diverse macrocycles that may have been missed. Furthermore, structure–activity relationships (SAR) were carried out by performing amino acid mutation scans to find specific regions of the sequences that were desirable for binding or tolerant to changes. To understand how the macrocycle binds to GIP-R, molecular dynamics simulations were performed using a representative example. The conformation of the macrocycle was found to agree well with the observations that had been determined from SAR study, including the tolerance for a Pro residue only in a region that adopts a hairpin turn.

Another benefit of the SAR and structural studies as part of the workflow was to determine which areas of the macrocycle may be amenable to functionalisation or mutation to other amino acids to improve important pharmacokinetic properties such as serum half-life and aqueous solubility. Addition of an albumin binding moiety at the solvent-exposed N-terminus, alongside charged residues to aid solubility, led to the design of optimised cyclic peptide lead compounds that showed excellent stability *in vivo*, several hours longer than that of semaglutide, a linear peptide used in the clinic to treat type 2 diabetes.

Overall, the process developed by Kawamura *et al.* provides a robust method to scan the hits generated from an mRNA display library and convert them into lead compounds for further development as therapeutics. The high-throughput clustering technique used allows for separation of non-functional hits and discovery of strongly binding macrocycles that may not have been found by simply searching for the most abundant sequences generated in the library. This methodology can now be applied to other drug discovery programs using mRNA display to scan the chemical space generated in a more efficient manner.

Aside from their stability in serum, one question that remains unknown from the study is how bioavailable are the molecules that were found here? Given the struggles associated with this class of molecule, it is abundantly clear that the major challenge associated with cyclic peptide therapeutics is no longer their discovery.^14^ Rather, the focus must shift to finding new tools that enable the rapid translation of these lead compounds into viable medicines.^15–17^ In fact, since mRNA display can feasibly load any unnatural building block onto tRNA for its incorporation into the library, perhaps new fragments must be sought that can lead to the development of emergent physicochemical properties alongside the high potency and selectivity that can already be achieved.

Nevertheless, ∼85% of the human proteome is considered “undruggable” to small molecules which do not have large enough size to bind to protein surfaces.^18^ Since mRNA display and other library-based technologies can be used to screen any protein or PPI of interest, there is huge potential for cyclic peptides and other macrocycles that can access this space to make a difference in many therapeutic areas.^19^ Future developments to the workflow of these techniques that ease the discovery process and make fuller use of large data sets, as demonstrated by Kawamura *et al.*, will be of high importance.

## Author contributions

G. J. S. wrote the manuscript with input from A. K. Y.

## Conflicts of interest

There are no conflicts of interest to declare.

## Supplementary Material
